# Baroreceptor reflex control of heart rate in angiotensin type 1A receptor knockout mice

**DOI:** 10.1002/phy2.171

**Published:** 2013-11-26

**Authors:** Yan-Ting Choong, Clement Menuet, Nikola Jancovski, Andrew M Allen

**Affiliations:** Department of Physiology and Florey Institute of Neuroscience and Mental Health, The University of MelbourneVictoria, 3010, Australia

**Keywords:** Angiotensin II, arterial baroreceptor reflex, blood pressure, cardiopulmonary baroreceptor reflex, isoflurane

## Abstract

The baroreceptor reflex dampens the short-term fluctuations in blood pressure by feedback modulation of heart rate (HR) and vascular resistance. Impairment of this reflex has been observed in hypertension and heart failure. Angiotensin II, a blood borne hormone, acts via its type 1A receptor to attenuate the baroreceptor reflex and this reflex is reported to be dramatically altered in angiotensin type 1A receptor knockout mice. This study sought to further investigate changes in the arterial and cardiopulmonary baroreceptor reflex control of HR in angiotensin II type 1A receptor knocked out mice. In artificially ventilated, isoflurane anesthetized mice, the arterial and cardiopulmonary baroreceptor reflexes were activated via injection or slow infusions, respectively, of phenylephrine and sodium nitroprusside through the jugular vein. We observed no impairment of either the arterial or cardiopulmonary baroreceptor reflex control of HR in angiotensin type 1A receptor knockout mice.

## Introduction

Arterial and cardiopulmonary baroreceptors are stretch-sensitive sensory afferent neurons that monitor blood vessel stretch in select vascular beds. In response to altered vascular stretch, these afferents induce a negative reflex loop that modulates autonomic and neuroendocrine function to ensure constant perfusion, particularly of the brain, and to dampen fluctuations in arterial blood pressure (BP). A key component of this reflex is the baroreceptor-heart rate (HR) reflex which induces rapid HR responses to alterations in BP.

There are three major sites of peripheral baroreceptor afferent terminals which because of their location, convey different sensory information related to the cardiovascular system. The arterial baroreceptors located in the carotid sinus and aortic arch monitor systemic and brain-directed arterial perfusion. The cardiopulmonary baroreceptors located within heart and large systemic veins detect venous pressure to primarily provide information about blood volume. It is proposed that the contribution of these reflex pathways to the baroreceptor-HR response can be studied using different methods to increase BP. Slow rises in BP tend to activate the arterial baroreceptors whereas rapid rises activate both arterial and cardiac baroreceptor pathways (Faris et al. 1980).

Following baroreceptor denervation, animals show dampened HR variability and an increase in BP variability. This demonstrates the importance of baroreceptor reflex in preventing acute fluctuations in BP (Cowley et al. 1973; Soares et al. 2006). The impairment of this reflex had been reported in many cardiovascular diseases such as hypertension (Timmers et al. 2004), stroke (Sykora et al. 2009), and heart failure (De Ferrari et al. 1995). A reduction in baroreceptor function is also shown to be a predictor of cardiac mortality (La Rovere et al. 1998).

Angiotensin II (Ang II) acts via the type 1A receptor (AT_1A_R) to exert multiple effects on the cardiovascular system, including attenuation of the baroreceptor reflex (Ackermann et al. 1989; Boscan et al. 2001). The AT_1A_R is expressed at multiple sites throughout the baroreceptor reflex pathway (Allen et al. 1998), including in the sensory afferent neurons and their terminals in the nucleus of the solitary tract (NTS) (Lewis et al. 1986) and intrinsic NTS neurons. Microinjection of Ang II into the NTS inhibits the baroreceptor reflex (Campagnole-Santos et al. 1988), whereas AT_1_R antagonist microinjection in the NTS improves baroreceptor sensitivity in models of hypertension (Matsumura et al. 1998). In addition, AT_1_R activation in the NTS plays an important role in the baroreceptor inhibition that occurs during development to allow BP to rise to adult levels (Kasparov et al. 1998). Consequently, the observation by Gembardt and colleagues that AT_1A_R knockout (AT_1A_R-KO) mice exhibit tachycardia, rather than the expected bradycardia, in response to a pharmacologically induced increase in BP seemed important (Gembardt et al. 2008). This is in contradiction to the observation that conscious AT_1A_R-KO mice do not exhibit altered baroreflex sensitivity (BRS) (Davern et al. 2009). Such gross impairment of the baroreceptor reflex suggested that the AT_1A_R plays a key role in either the development or ongoing function of the baroreceptor-HR reflex under basal conditions.

Consequently, the aim of the current investigation was to examine the baroreceptor-HR reflex more fully to determine whether arterial and/or cardiopulmonary baroreceptor modulation of HR is altered in AT_1A_R-KO mice.

## Materials and Methods

### Animals

All experimental procedures were approved by the Animal Experimentation and Ethics Committee of the University of Melbourne and were conducted in accordance with the National Health and Medical Research Council of Australia “Guidelines to promote the well-being of animals used for scientific purposes”. The mice were maintained under 12:12 light: dark cycle with ad libitum access to food and water. The experiments examined two homozygous strains that were initially obtained from Prof T. Walther that are maintained at the University of Melbourne Animal Facility (Chen et al. 2010). One strain (AT_1A_R-KO) had targeted deletion of the *Agtr1A* gene locus which encodes the AT_1A_R, and the other a wild-type strain back-crossed on C57Bl/6 background (WT). Experiments were performed on male mice at either 4 or 7 months of age.

### BP measurement

In all groups of mice, anesthesia was induced by inhalation of 3% isoflurane (Delvet Isoflurane, Therapon Pty. Ltd., Melbourne, Australia) in O_2_ until loss of the pedal withdrawal reflex. A surgical plane of anesthesia was maintained by delivery of 2% isoflurane in 100% O_2_ initially via a nose cone. Following tracheotomy, it was delivered via a rodent ventilator (SAR-830/P Ventilator, CWE, Ardmore, PA) at 100 rpm × 0.30 mL. End-tidal CO_2_ was measured (CAPSTAR-100 carbon dioxide analyzer, CWE) and maintained between 2.5% and 3.5%. Higher end-tidal CO_2_ concentrations induced strong respiratory efforts against the ventilated with pronounced, transient changes in BP. Throughout the protocol, body temperature was monitored with a rectal probe and maintained at 37.5°C with a heat pad (TC-1000 temperature controller, CWE).

The left carotid artery was cannulated with a polyethylene (PE) catheter (OD 0.97 mm, ID 0.58 mm) that was pulled over hot air to create a taper with an outer tip diameter of 0.3–0.35 mm. Care was taken not to injure the vagus nerve during cannulation. The BP was continuously monitored with a disposable pressure transducer (BD Becton Dickinson, North Ryde, New South Wales, Australia.) filled with heparinized sterile saline. The left jugular vein was cannulated with a PE catheter (OD 0.61 mm, ID 0.28 mm) with a tapered tip. All parameters were digitized and recorded using a CED Micro1401 interface (CED, Cambridge, U.K.). The BP signal was used to derive mean arterial pressure (MAP) and HR using Spike 2 (CED). After completion of the surgical preparation, a stabilization period of 10 min was allowed before a recording of basal BP was made.

### Ramp baroreflex technique (Bolus injections)

Initially, the BP and HR response to multiple i.v. injections of phenylephrine (Phe) (20–50 *μ*L, 25 *μ*g/mL. Sigma Aldrich, Sydney, Australia) and sodium nitroprusside (SNP) (10–50 *μ*L, 1 ng/mL, Sigma Aldrich) was determined to assess the cardiopulmonary baroreceptor-HR reflex. The bolus injections were given randomly with a recovery period of 10–15 min between each treatment. The maximum MAP and HR changes from baseline were quantified. The BRS was calculated in two ways. Firstly, the ΔHR response to changes in MAP by −10.6 ± 2.1, 20 ± 2.4, 29.3 ± 2.2, and 38.3 ± 3.0 mmHg were quantified. Secondly, to standardize the baroreflex response of the HR for different MAP changes, the BRS of HR was calculated as a ratio of ΔHR/ΔMAP.

### Steady-state baroreflex technique (Slow infusion)

After an additional recovery period of 20 min, Phe (25 *μ*g/mL) and SNP (1 ng/mL) were infused to assess the arterial baroreceptor-HR reflex. A step-wise increase in rate of infusion of the drugs was performed at 0.35, 0.87, 1.71, 3.46, 7.2, and 15.0 mL/h for 30 sec at each infusion rate before a further increase in the rate of infusion. Changes in of HR were measured at each progressive 5 mmHg change from baseline. The sensitivity of baroreflex control of HR was determined by performing linear regression analysis at the linear part of the MAP/HR response curve (Graph Pad Prism 5 for windows, Version 5.03, Graph Pad Software, CA).

### Statistics

All results are reported as mean ± standard deviation. For most comparisons, data were analyzed using two-way analysis of variance (ANOVA) (Graph Pad Prism 5). *P* values <0.05 were considered statistically significant. To compare linear regression of BRS between groups, 95% confidence intervals were used. Capped error bars denote standard deviations. Uncapped error bars denote 95% confidence intervals of the mean.

## Results

Baseline levels of BP and HR prior to any experimental procedures are shown on Table 1. As reported previously, BP was significantly reduced in AT_1A_R-KO mice at each age. There was no significant effect of age on basal BP and HR. A genotype effect (*P* < 0.05) on basal HR was observed with a reduction in HR in 7-month AT_1A_R-KO compared to their WT counterparts (465 ± 68 bpm vs. 551 ± 53 bpm).

**Table 1:** Baseline resting MAP and HR of the different mouse groups during isoflurane anesthesia

Genotype	Age (months)	Number	MAP (mmHg)	HR (bpm)
AT_1A_R-KO	4.2 ± 0.4	10	47 ± 7***	524 ± 47
AT_1A_R-KO	7.9 ± 1.5	9	51 ± 5***	465 ± 68*
AT_1A_R-WT	3.8 ± 0.4	9	89 ± 16	518 ± 39
AT_1A_R-WT	7.2 ± 1.2.	13	86 ± 15	551 ± 53

Values are mean ± SD. MAP, mean arterial pressure; HR, heart rate.

*** *P* < 0.001 versus WT counterparts;

* *P* < 0.05 versus WT counterparts.

### Response to intravenous injections of Phe and SNP

**Figure 1:**
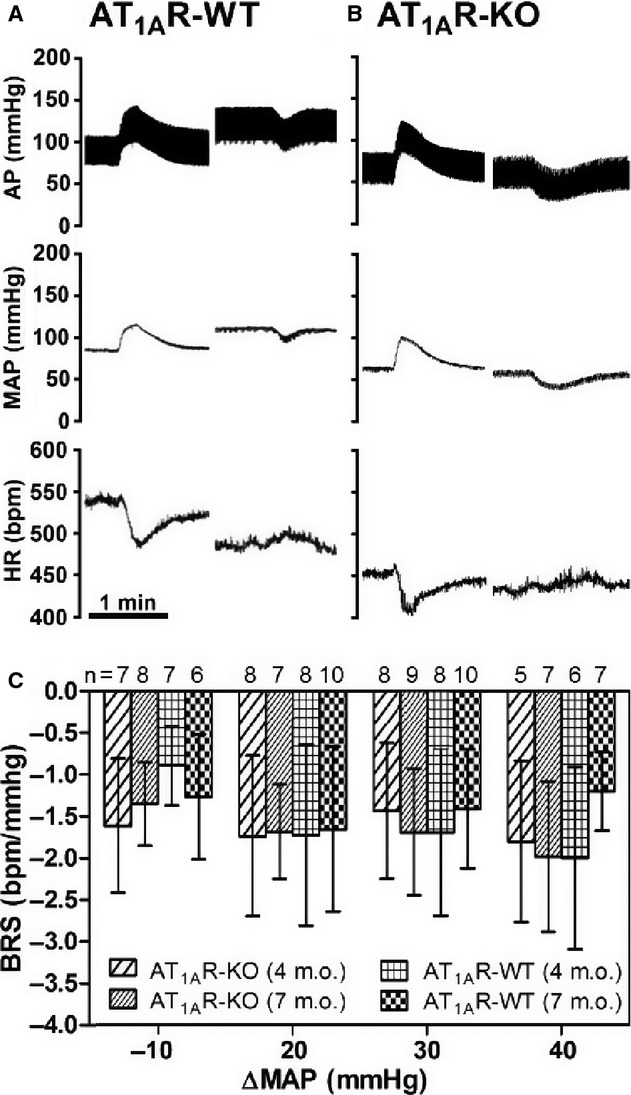
Representative traces of arterial pressure (AP), mean arterial pressure (MAP), and heart rate (HR) during i.v. injections of Phe (right) and SNP (left) in a 4-month-old AT_1A_R-WT (A) and an AT_1A_R-KO (B) mouse. The histogram (C) shows changes in cardiopulmonary baroreflex sensitivity (BRS) in response to bolus injections of Phe and SNP in the four groups of mice. The number of observations in each group is denoted above each bar of the histogram.

Intravenous bolus injections of Phe and SNP induced rapid changes in BP and HR that were similar in both WT and AT_1A_R-KO mice (Fig. 1A and B). This was associated with a decrease in HR in all animals of both genotypes. There was no effect of genotype or age on the HR response to a MAP change of any magnitude or in the sensitivity index of the baroreflex (Fig. 1C).

### Response to ramp infusions of Phe and SNP

**Figure 2:**
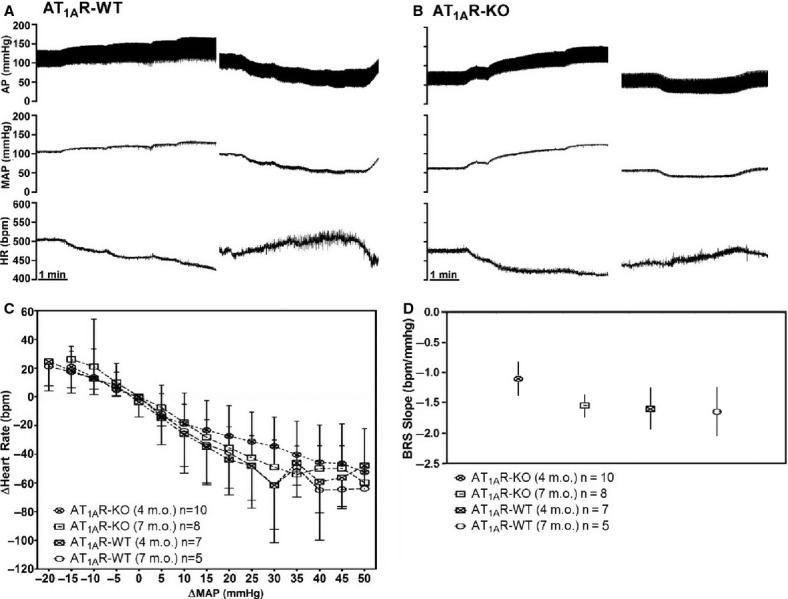
Representative traces from a 4-month-old AT_1A_R-WT (A) and an AT_1A_R-KO (B) mouse showing arterial pressure (AP), mean arterial pressure (MAP), and heart rate (HR) during slow i.v. infusions of Phe (right) and SNP (left). Changes in HR mediated by the arterial baroreflex in response to slow infusions of Phe and SNP in the four groups of mice are shown (C). Baroreflex sensitivity, derived from the linear portion of the MAP/HR relationship, is shown for each group in the histogram (D). There were no significant differences between animal groups for the baroreflex sensitivity. (AT_1A_R-KO 4 m.o. BRS = −1.099 bpm/mmHg, 95% CI [−1.376, −0.8226]; AT_1A_R-KO 7 m.o. BRS = −1.544 bpm/mmHg, 95% CI [−1.734, −1.354]; AT_1A_R-WT 4 m.o. BRS = −1.595 bpm/mmHg, 95% CI [−1.942, −1.247]; AT_1A_R-WT 7 m.o. BRS = −1.644 bpm/mmHg, 95% CI [−2.048, −1.240]). Capped error bars denote standard deviations. Uncapped error bars denote 95% confidence intervals of the mean.

Intravenous ramp infusions of SNP and Phe evoked MAP changes ranging from −20 mmHg to +40 mmHg from baseline that were similar in magnitude and time course in WT and AT_1A_R-KO mice (Fig. 2A and B). In all animals, HR and MAP is asymptotic with plateaus at both high and low levels MAP and a linear relationship over intermediate MAP levels (Fig. 2C). Analysis of the slope of the linear portion of the MAP/HR response showed that there were no significant differences between animal groups for BRS slope (AT_1A_R-KO 4 m.o. BRS = −1.099 bpm/mmHg, 95% CI [−1.376, −0.8226]; AT_1A_R-KO 7 m.o. BRS = −1.544 bpm/mmHg, 95% CI [−1.734, −1.354]; AT_1A_R-WT 4 m.o. BRS = −1.595 bpm/mmHg, 95% CI [−1.942, −1.247]; AT_1A_R-WT 7 m.o. BRS = −1.644 bpm/mmHg, 95% CI [−2.048, −1.240]) (Fig. 2D). A total of 800–850 *μ*L of fluid was administered over 3–4 h of experimentation.

## Discussion

Based upon the observations included in this study, we conclude that the baroreflex-mediated regulation of HR in AT_1A_R-KO is not significantly different to WT. Neither method of baroreceptor stimulation, which we employed to examine the relative involvement of arterial and cardiopulmonary baroreceptors, showed any difference in response between AT_1A_R and WT mice. There was also no difference in the baroreceptor- HR response between AT_1A_R-KO and WT mice when examined at older ages (7 months vs. 4 months).

Our results are consistent with observations in conscious AT_1A_R-KO mice (Davern et al. 2009) that AT_1A_R-KO mice do not have altered BRS when examined by measurement of spontaneous changes in the BP/HR relationship. Our observations differ markedly from those reported by Gembardt et al. (2008) in anesthetized mice which showed that the baroreceptor reflex was grossly inverted in AT_1A_R-KO mice. They observed tachycardia in response to intravenous injection of Phe in AT_1A_R-KO mice whereas WT, AT_1B_R-KO, and AT_2_R-KO mice showed the expected bradycardia (Gembardt et al. 2008). The reason for this marked difference between the studies in AT_1A_R-KO mice remains unclear. We initially examined 4-month-old mice whereas Gembardt et al. reported observations from mice at 7 months. Hence, we examined this slightly older age but found no difference between 4-month and 7-month-old mice of either genotype.

It is most likely that the choice of anesthetic explains the different baroreceptor reflex responses observed. Gembardt and colleagues used pentobarbitone anesthesia whereas the observations reported in this study used isoflurane. We attempted to repeat these studies in pentobarbital anesthetized mice, but found it difficult to achieve stable BP and consistent baroreceptor reflex responses under these conditions. Previous studies have reported that pentobarbital anesthesia can dampen baroreceptor responses in rodents (Shimokawa et al. 1998; Ma et al. 2002). The bradycardia in response to electrical stimulation of aortic depressor nerve is completely abolished in pentobarbital anesthetized mice, thus demonstrating an impairment of central baroreflex processing by this anesthetic (Ma et al. 2002). In rats, pentobarbital administration decreased the gain of baroreceptor reflex control of both HR and renal sympathetic nerve activity (Shimokawa et al. 1998). Comparing the current study with that of Gembardt also shows that pentobarbital anesthesia reduces resting BP and basal HR substantially more than isoflurane, in both WT and AT_1A-_R-KO mice. It is quite likely that this alteration in basal BP and HR would affect BRS. Pentobarbital administration is known to suppress plasma catecholamine levels (Baum et al. 1985) suggesting impairment of sympatho-adrenal function. We conclude that cardiovascular data from pentobarbital-anesthetized mice should be interpreted with caution.

Although isoflurane depresses the central and efferent component of the baroreflex, this appears to be compensated by a hypersensitization of the sinus nerve activity to changes in sinus pressures (Seagard et al. 1983). In dogs anesthetized with surgical levels of isoflurane (<2.6%), baroreceptor regulation of HR is similar to that observed in conscious dogs (Seagard et al. 1983). Similar observations have been reported in rats (Lee et al. 2002) and both these studies indicate that the dampening effect of isoflurane on maximal reflex tachycardia is primarily due to suppression of sympathetic components of the baroreflex arc (Seagard et al. 1983; Lee et al. 2002). Overall, the literature supports the view that isoflurane is a superior anesthetic in studies assessing baroreflex function.

Given the markedly reduced resting BP in the AT_1A_R-KO, which would be close to the threshold for baroreceptor activation in WT mice, it is perhaps surprising that there is no obvious change in baroreceptor reflex sensitivity in these mice. We suggest that this is due to the reflex being reset to a different operating set-point pressure, as has been described previously. In conclusion, our observations indicate that there is no obvious alteration in arterial and cardiopulmonary baroreceptor reflex function in AT_1A_R-KO mice.
